# Supplementary material for: Modeling and therapeutic targeting of inflammation-induced hepatic insulin resistance using human iPSC-derived hepatocytes and macrophages
Source: Nat Commun. 2023 Jul 3;14:3902. doi: 10.1038/s41467-023-39311-w (PMC10318012; doi:10.1038/s41467-023-39311-w)
Supplement: Supplementary file 1 — Supplementary information [file 41467_2023_39311_MOESM1_ESM.pdf]

**Supplementary information**

Modeling and therapeutic targeting of inflammation-induced hepatic insulin resistance using human iPSC-derived hepatocytes and macrophages

Marko Groeger, Koji Matsuo, Emad Heidary Arash, Ashley Pereira, Dounia Le Guillou, Cindy Pino, Kayque A. Telles-Silva, Jacquelyn J. Maher, Edward C. Hsiao, Holger Willenbring

1. Included in this file: Supplementary Figs. 1-8, Supplementary Tables 1-4

2. Additional information: Supplementary Data 1

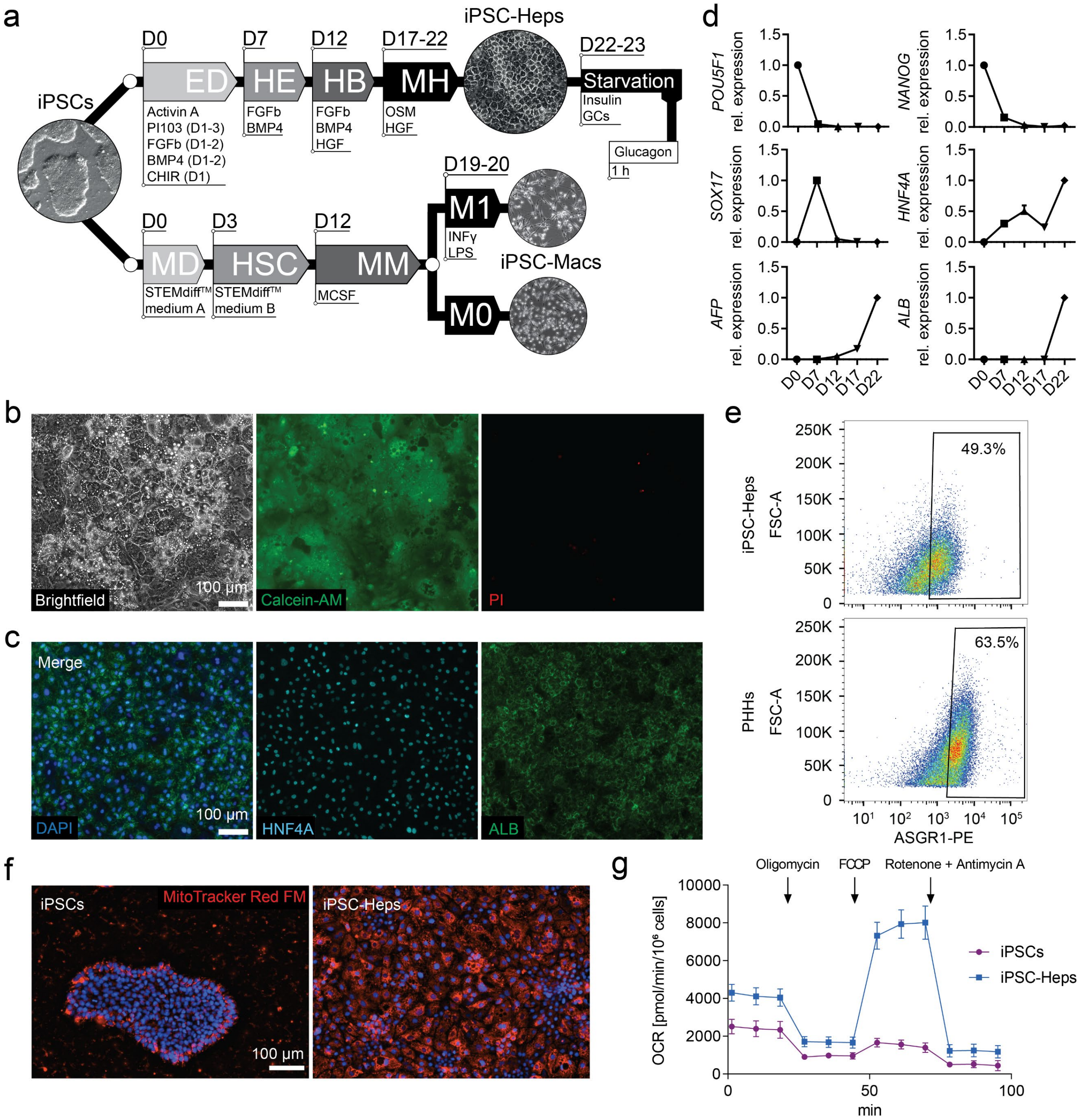


**Supplementary Fig. 1 | Generation of iPSC-Heps and iPSC-Macs. a** Overview of protocol for generation of iPSC-Heps and iPSC-Macs highlighting developmental stages and differentiation-promoting factors. D, day; ED, endoderm; HE, hepatic endoderm; HB, hepatoblast; MH, mature hepatocyte; MD, mesoderm; HSC, hematopoietic stem cell; MM, mature macrophage; GCs, glucocorticoids. **b** Representative live imaging of iPSC-Heps stained with the viability markers Calcein-AM (viable, green) and propidium iodide (PI; dead, red) at the end of the differentiation protocol. n = 3. **c** Representative immunofluorescence of HNF4A and albumin (ALB) in iPSC-Heps. n = 3. **d** Time course of gene expression analysis of pluripotency (*POU5F1*, *NANOG*), endoderm (*SOX17*) and hepatocyte (*HNF4A*, *AFP*, *ALB*) markers in iPSC-Heps. Data are mean ± SD; n = 3. **e** Representative flow cytometry analysis of the hepatocyte differentiation marker ASGR1 in iPSC-Heps and freshly thawed PHHs. iPSC-Heps: n = 3, PHHs: n = 1. **f** Representative live imaging of mitochondria (MitoTracker Red FM) in iPSCs and iPSC-Heps. n

= 3. **g** Mitochondrial respiration (oxygen consumption rate, OCR) in iPSCs and iPSC-Heps. Data are mean ± SD; iPSCs: n = 4, iPSC-Heps: n = 10. Source data are provided as a Source Data file.

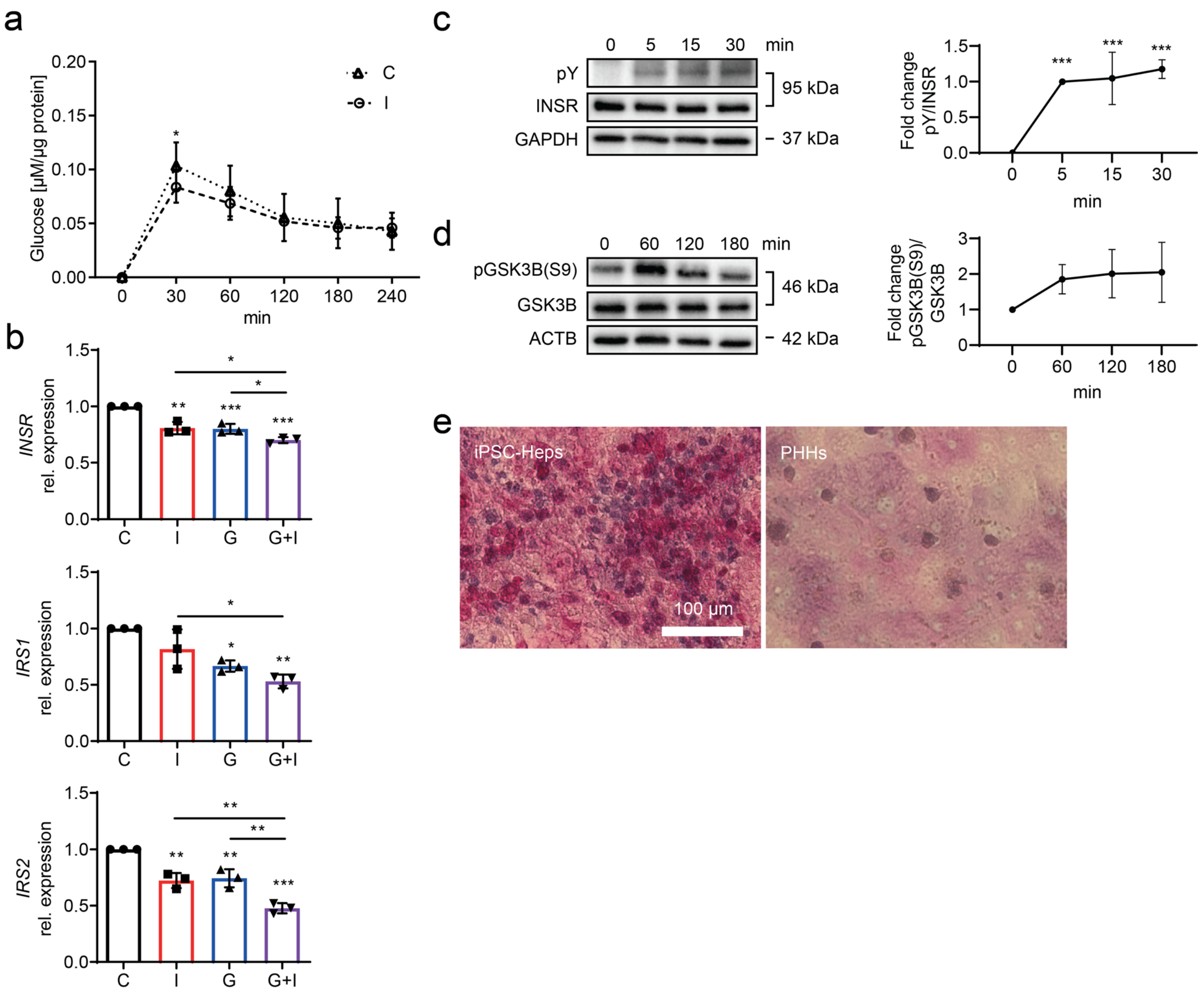


**Supplementary Fig. 2 | Insulin and glucagon effects on glucose metabolism in iPSC-Heps. a** Time course of analysis of glucose release by iPSC-Heps into glucose-free media after insulin (I) and no insulin (C, control). Data are mean ± SD; n = 6, two-way ANOVA (two-stage step-up method of Benjamini, Krieger and Yekutieli), *$P$ < 0.05 vs. I at indicated timepoints. **b** Gene expression analysis in iPSC-Heps after 4 h of no hormones (C, control), insulin (I), glucagon (G) or 2 h of glucagon followed by 2 h of insulin (G+I). Data are mean ± SD; n = 3, one-way ANOVA (Tukey's test), *$P$ < 0.05, **$P$ < 0.01 and ***$P$ < 0.001 vs. C or between indicated conditions. **c** Time course of western blot analysis of INSR autophosphorylation in iPSC-Heps after insulin. Data are mean ± SD; n = 3, one-way ANOVA (Dunnett's test), ***$P$ < 0.001 vs. 0 min. **d** Time course of western blot analysis of GSK3B phosphorylation in iPSC-Heps after insulin. Data are mean ± SD; n = 3, one-way ANOVA (Dunnett's test). **e** Representative Periodic Acid Schiff staining of iPSC-Heps at the end of the differentiation protocol and PHHs 48 h after plating (glycogen is red/pink, nuclei are purple/unstained). n = 3. Source data are provided as a Source Data file.

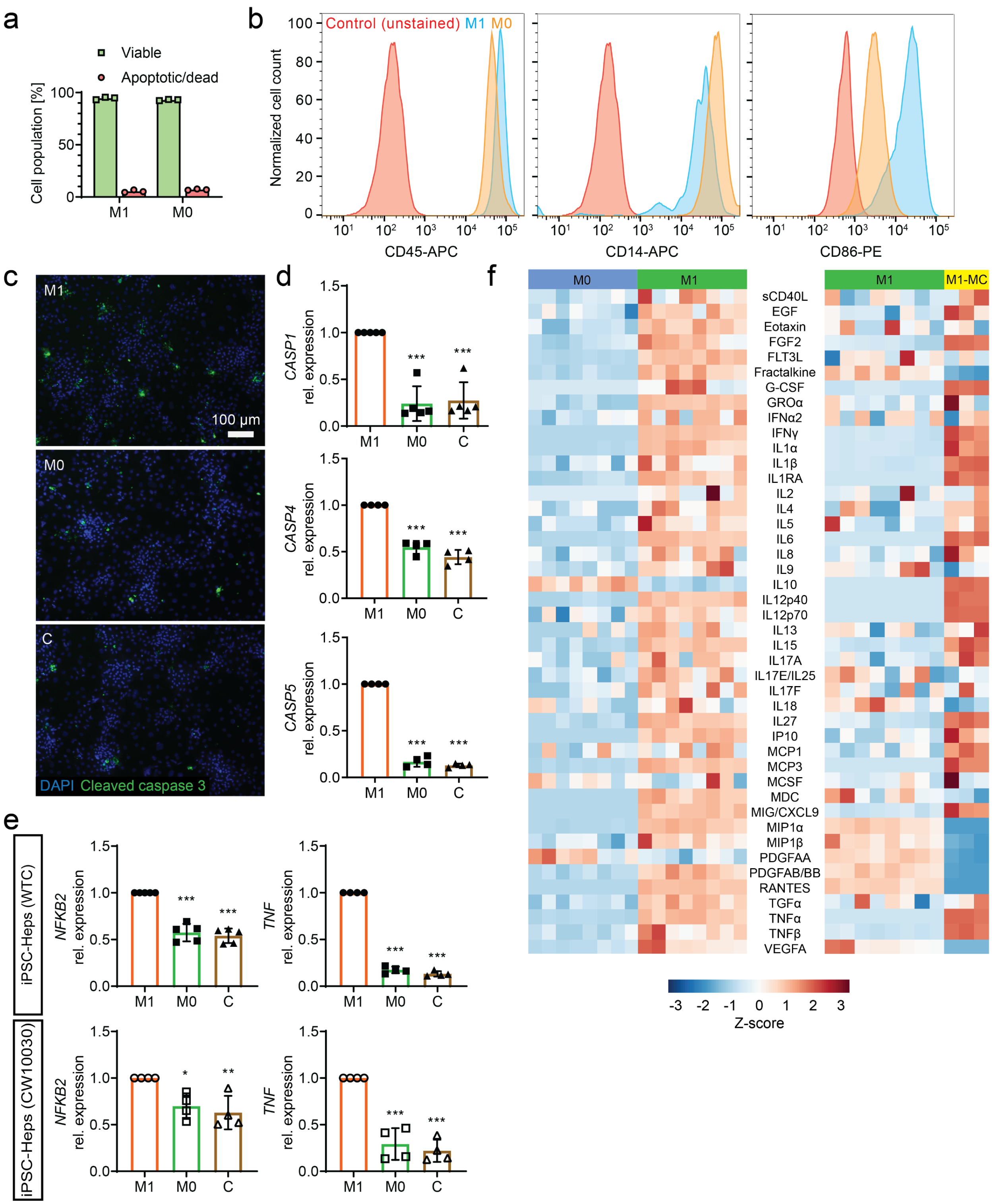


**Supplementary Fig. 3 | Inflammation of iPSC-Heps co-cultured with M1 or M0 iPSC-Macs.** **a, b** Flow cytometry analysis of viability (**a**) and hematopoietic (CD45) and monocytic (CD14) differentiation and macrophage polarization (CD86) markers (**b**) in M1 or M0 iPSC-Macs. Data are mean ± SD; n = 3. **c-f** Representative immunofluorescence of cleaved caspase 3 particles in iPSC-Heps (**c**), caspase gene expression analysis in iPSC-Heps (*CASP1*: n = 5, *CASP4* and

*CASP5*: n = 4) (**d**), inflammatory gene expression analysis in WTC and CW10030 iPSC-Heps (WTC *NFKB2*: n = 5, WTC *TNF* and CW10030 *NFKB2* and *TNF*: n = 4) (**e**) and comparative analysis of cytokine and chemokine release into media (M0 and M1: n = 8, M1-MC: n = 3) (**f**) after 24-h co-culture of iPSC-Heps with M1 or M0 iPSC-Macs, in iPSC-Hep mono-culture (C, control) or in M1 iPSC-Mac monoculture (M1-MC). Data are mean ± SD; one-way ANOVA (Dunnett's test), *$P < 0.05$, **$P < 0.01$, ***$P < 0.001$ vs. M1. Source data are provided as a Source Data file.

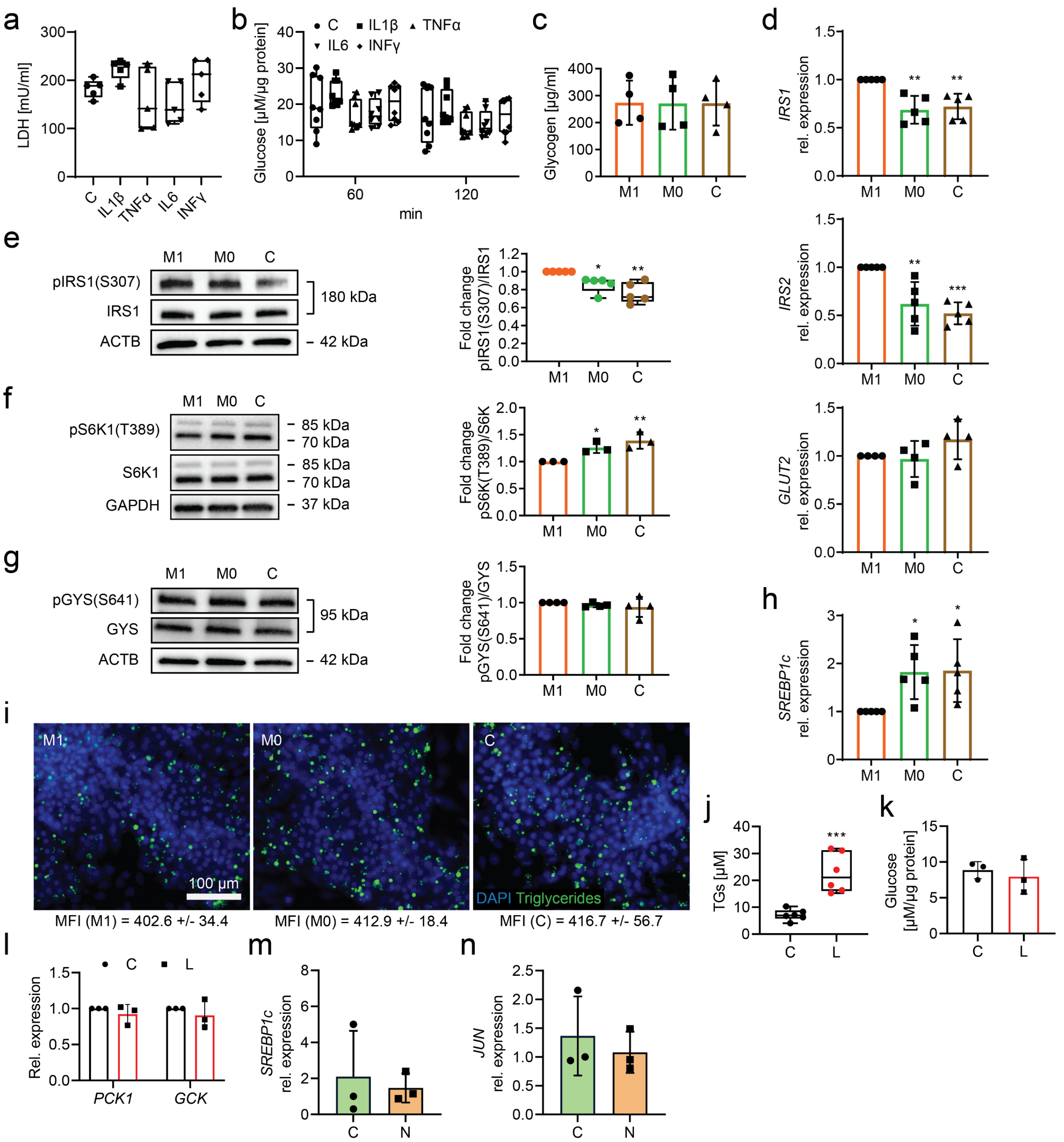


**Supplementary Fig. 4 | Glucose metabolism changes in iPSC-Heps co-cultured with M1 or M0 iPSC-Macs or accumulating lipid. a** Quantification of LDH release into media by iPSC-Heps treated with the indicated cytokines for 24 h. Data are mean ± SD; n = 5. **b** Quantification of glucose release into 1 mM glucose-containing media 2 h after insulin by iPSC-Heps treated with the indicated cytokines for 24 h. Data are mean ± SD; n = 8. **c** Quantification of glycogen in iPSC-Heps after 24-h co-culture of iPSC-Heps with M1 or M0 iPSC-Macs or in iPSC-Hep mono-culture (C, control). Data are mean ± SD; n = 4, one-way ANOVA (Dunnett's test). **d-g** Gene expression analysis in iPSC-Heps 1 h after insulin (*IRS1* and *IRS2*: n = 5, *GLUT2*: n = 4) (**d**), western blot analysis in iPSC-Heps of IRS1 phosphorylation 5 min after insulin (n = 5) (**e**), western blot analysis in iPSC-Heps of S6K1 phosphorylation 30 min after insulin (n = 3) (**f**) and

western blot analysis in iPSC-Heps of GYS phosphorylation 2 h after insulin (n = 4) (**g**) after 24-h co-culture of iPSC-Heps with M1 or M0 iPSC-Macs or in iPSC-Hep mono-culture (C, control). Data are mean ± SD; one-way ANOVA (Dunnett's test), **P* < 0.05, ***P* < 0.01 and ****P* < 0.001 vs. M1. **h** Gene expression analysis in iPSC-Heps 1 h after insulin after 24-h co-culture of iPSC-Heps with M1 or M0 iPSC-Macs or in iPSC-Hep mono-culture (C, control). Data are mean ± SD; n = 5, one-way ANOVA (Dunnett's test), **P* < 0.05 vs. M1. **i** Representative triglyceride staining in iPSC-Heps after 24-h co-culture of iPSC-Heps with M1 or M0 iPSC-Macs or in iPSC-Hep mono-culture (C, control). MFI values are mean ± SD; n = 3. MFI, mean fluorescence intensity of BODIPY 493/503. **j-l** Quantification of triglycerides (TGs) (n = 6) (**j**), quantification of glucose release into 1 mM glucose-containing media 2 h after insulin (n = 3) (**k**) and gene expression analysis 1 h after insulin (n = 3) (**l**) in iPSC-Heps after mono-culture without (C, control) or with 100 μM oleate and 100 μM palmitate (L, lipids) for 6 days. Data are mean ± SD; unpaired two-tailed Student's t test, ****P* < 0.001 vs. C. **m, n** Gene expression analysis in iPSC-Heps 1 h after insulin (n = 3) (**m**) and at the end of the differentiation protocol (n = 3) (**n**) comparing iPSC-Heps from 3 healthy (C, control) and 3 NASH (N) patients. Data are mean ± SD; unpaired two-tailed Student's t test. Source data are provided as a Source Data file.

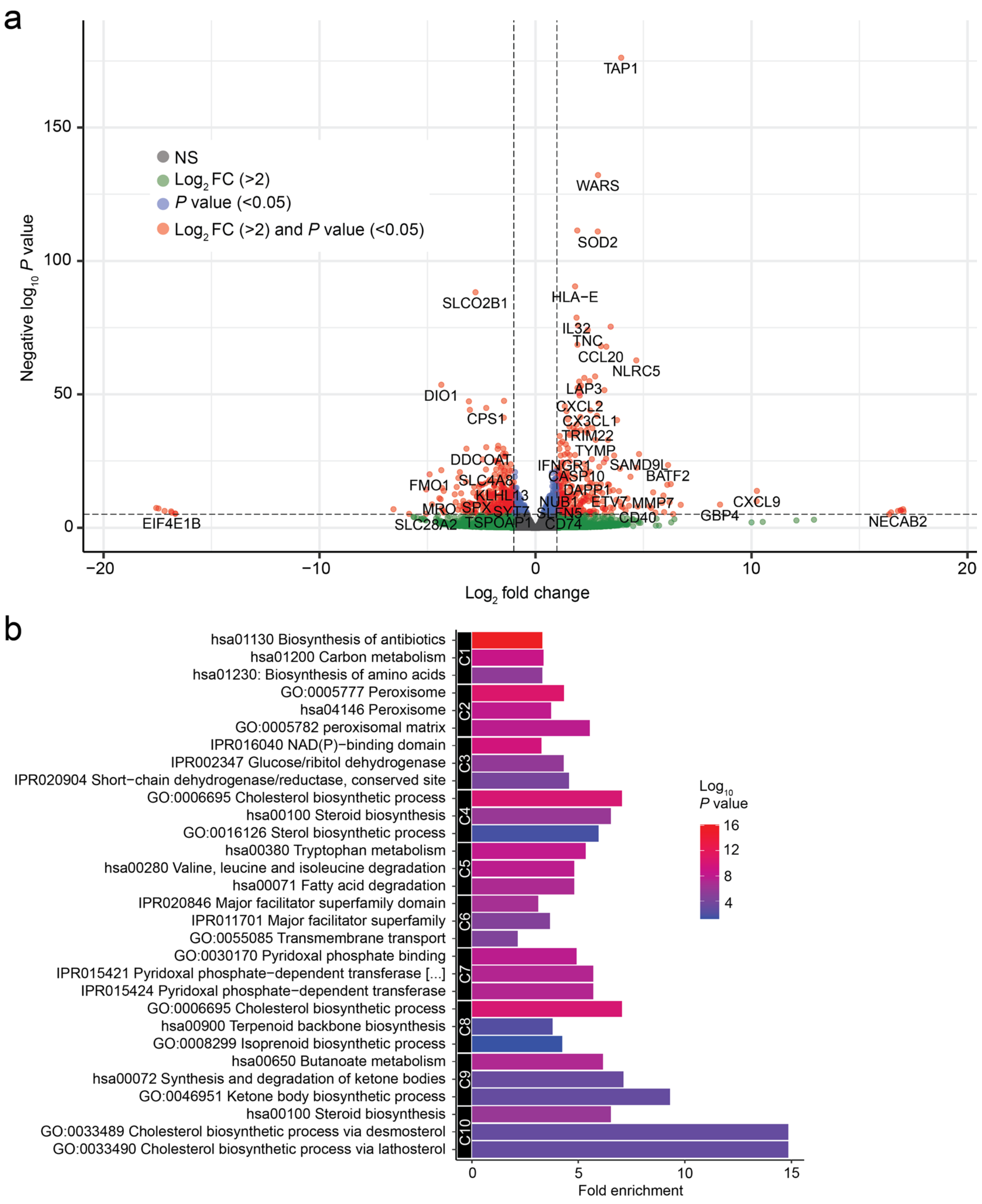


**Supplementary Fig. 5 | RNAseq analysis of iPSC-Heps co-cultured with M1 or M0 iPSC-Macs. a** Volcano plot of all differentially expressed genes with log2 fold change > 2, *P* value < 0.05 or both in iPSC Heps co-cultured with M1 or M0 iPSC-Macs. Horizontal line indicates *P* value < 0.05 and vertical lines mark log2 fold change of 2.0 and -2.0. n = 3, FDR-adjusted *P* value (*P* < 0.05) by Wald test. FC, fold change; NS, not significant. **b** Top 10 down-regulated pathway clusters enriched (Cluster Enrichment Score > 1.3) in the genes differentially expressed between iPSC Heps co-cultured with M1 or M0 iPSC-Macs identified using DAVID. Vertical axis represents enrichment fold values and horizontal axis shows the names of GO-BP, GO-MF and GO-CC terms and KEGG pathways. Node color indicates the enrichment significance, red represents higher significance. n = 3, FDR-adjusted *P* value (*P* < 0.05) by Wald test.

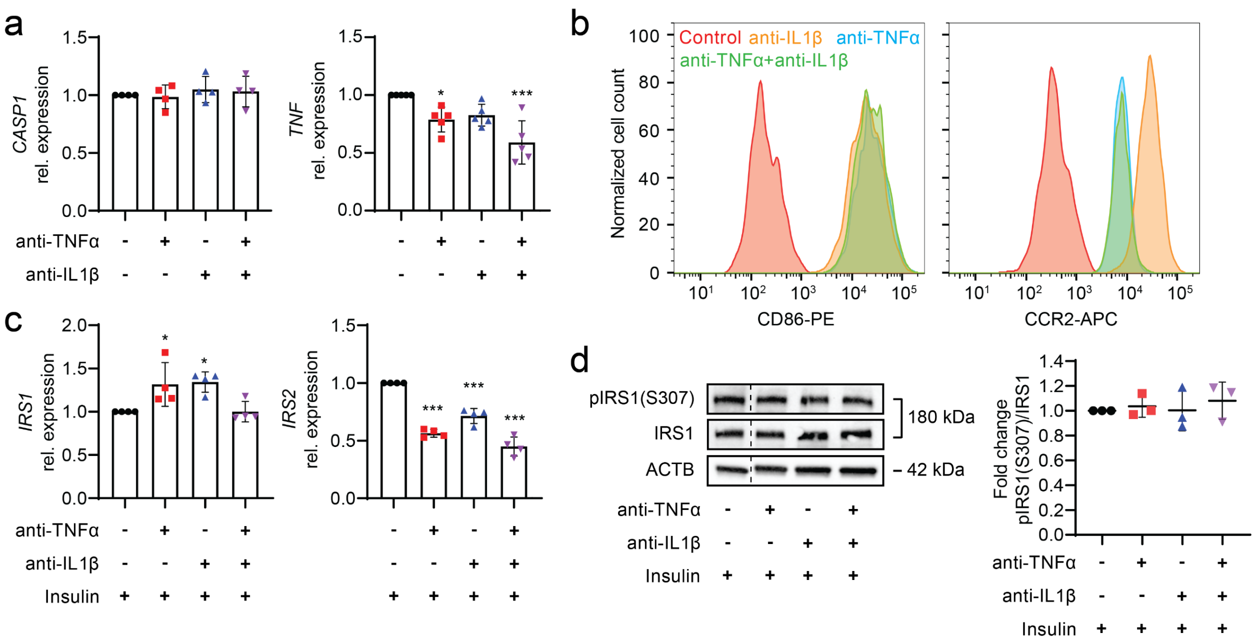


**Supplementary Fig. 6 | Effect of TNFα and/or IL1β neutralization on inflammation and glucose metabolism changes in iPSC-Heps co-cultured with M1 iPSC-Macs. a** Gene expression analysis in iPSC-Heps after 24-h co-culture with M1 iPSC-Macs and indicated antibody treatments. Data are mean ± SD; *CASP1*: n = 4, *TNF*: n = 5, one-way ANOVA (Dunnett's test), *$P$ < 0.05 and ***$P$ < 0.001 vs. no-antibody condition. **b** Flow cytometry analysis of CD86 and CCR2 in M1 iPSC-Macs after 24-h co-culture with iPSC-Heps and indicated antibody treatments. n = 3. **c**, **d** Gene expression analysis 1 h after insulin (n = 4) (**c**) and western blot analysis of IRS1 phosphorylation 5 min after insulin (n = 3) (**d**) in iPSC-Heps after 24-h co-culture with M1 iPSC-Macs and indicated antibody treatments. Data are mean ± SD; one-way ANOVA (Dunnett's test), *$P$ < 0.05 and ***$P$ < 0.001 vs. no-antibody condition. Source data are provided as a Source Data file.

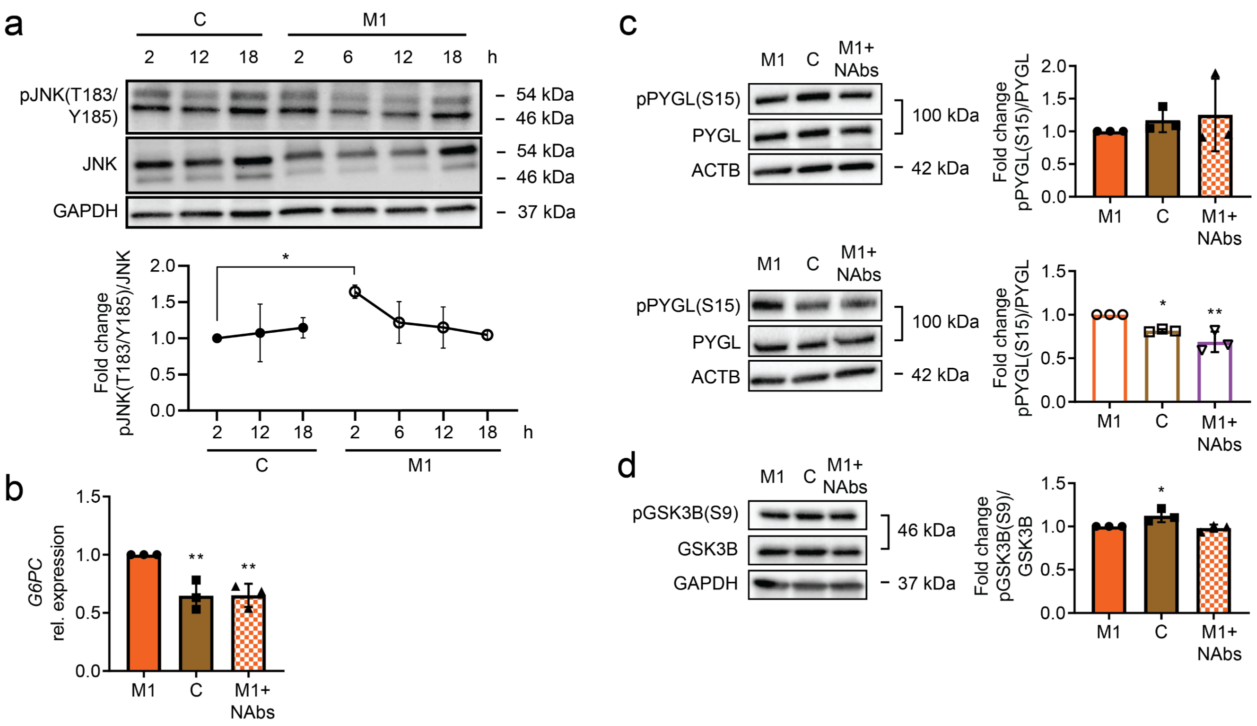


**Supplementary Fig. 7 | Effect of TNFα and/or IL1β neutralization on inflammation and glucose metabolism changes in PHHs co-cultured with M1 PHMs. a** Time course of western blot analysis of JNK phosphorylation in PHHs after co-culture with M1 PMHs or in PHH mono-culture (C, control). Data are mean ± SD; n = 3, one-way ANOVA (Tukey's test), *$P$ < 0.05. **b** Gene expression analysis 1 h after insulin after 24-h co-culture of PHHs with M1 or M0 PHMs or in PHH mono-culture (C, control). Data are mean ± SD; n = 3, one-way ANOVA (Dunnett's test), **$P$ < 0.01 vs. M1. **c** Western blot analysis of PYGL phosphorylation 30 min after insulin in hepatocytes after 24-h co-culture with M1 macrophages or in hepatocyte mono-culture (C, control) or after 24-h co-culture with M1 macrophages including TNFα and IL1β neutralizing antibodies (M1+NAbs). Primary cells, filled bars/symbols; CW10030 iPSC-derived cells, open bars/symbols. Data are mean ± SD; n = 3, one-way ANOVA (Dunnett's test), *$P$ < 0.05 and **$P$ < 0.01 vs. M1. **d** Western blot analysis of GSK3B phosphorylation 30 min after insulin in PHHs after 24-h co-culture with M1 PMHs or in PHH mono-culture (C, control) or after 24-h co-culture with M1 PHMs including TNFα and IL1β neutralizing antibodies (M1+NAbs). Data are mean ± SD; n = 3, one-way ANOVA (Dunnett's test), *$P$ < 0.05. Source data are provided as a source Data File.

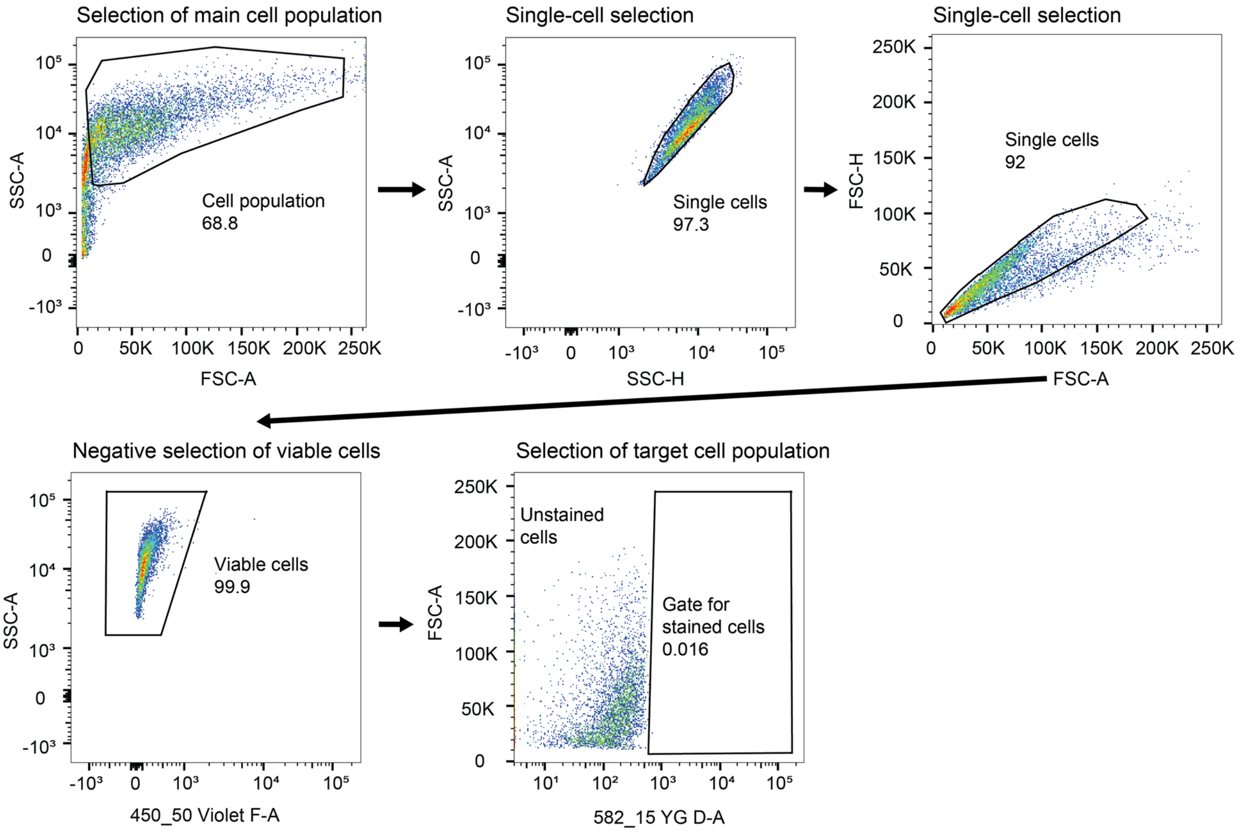


**Supplementary Fig. 8 | Gating strategy for flow cytometry analysis.** The main cell population was selected based on pulse area (-A) of forward scatter (FSC) and side scatter (SSC). Pulse height (-H) of FSC and SSC was used to select single cells from the main cell population. SYTOX green/red cell death staining was used to select viable cells. Gates for target cells expressing specific markers detected with fluorophore-conjugated antibodies were defined based on unstained cells. 450_50 Violet F-A and 582_15 YG D-A are flow cytometer-specific laser and filter set combinations for fluorophore detection.

**Supplementary Table 1 | Primers for qRT-PCR.**

| Gene | Direction | Sequence |
|---|---|---|
| *CASP1* | Forward | CTCAGGCTCAGAAGGGAATG |
| | Reverse | ACGCTGTACCCCAGATTTTG |
| *CASP4* | Forward | CAAGAGAAGCAACGTATGGCA |
| | Reverse | AGGCAGATGGTCAAACTCTGTA |
| *CASP5* | Forward | TTCAACACCACATAACGTGTCC |
| | Reverse | GTCAAGGTTGCTCGTTCTATGG |
| *G6PC* | Forward | ACTGGCTCAACCTCGTCTTTA |
| | Reverse | CGGAAGTGTTGCTGTAGTAGTCA |
| *GCK* | Forward | CCGCAAGCAGATCTACAACA |
| | Reverse | AGCTTGTACACGGAGCCATC |
| *GLUT2* | Forward | TTGGTGTGATCAATGCACCT |
| | Reverse | GCCACAGTCTCTTCCTCAGC |
| *INSR* | Forward | CATCCGGGGATCACGACTG |
| | Reverse | ATCAGGTTGTAGAGGCCGAGT |
| *IRS1* | Forward | CAAGACCATCAGCTTCGTGA |
| | Reverse | AGAGTCATCCACCTGCATCC |
| *IRS2* | Forward | CGGTGAGTTCTACGGGTACAT |
| | Reverse | TCAGGGTGTATTCATCCAGCG |
| *NFKB2* | Forward | ATGGAGAGTTGCTACAACCCA |
| | Reverse | CTGTTCCACGATCACCAGGTA |
| *PCK1* | Forward | TCATTTAAGGGCCATCAACC |
| | Reverse | CTCATCAATGCCTTCCCAGT |
| *RPLP0* | Forward | GCAGATCCGCATGTCCCTT |
| | Reverse | TGTTTTCCAGGTGCCCTCG |
| *SREBP1c* | Forward | CCATGGATTGCACTTTCGAA |
| | Reverse | GGCCAGGGAAGTCACTGTCTT |
| *TNF* | Forward | CCTCTCTCTAATCAGCCCTCTG |
| | Reverse | GAGGACCTGGGAGTAGATGAG |

**Supplementary Table 2 | Antibodies.**

| Antibody | Company | Catalogue # | Dilution |
|---|---|---|---|
| Immunofluorescence | | | |
| Goat anti-Albumin | Bethyl Lab. | A80-229A | 1:500 |
| Rabbit anti-Cleaved Caspase 3 | Cell Signaling | 9661s | 1:400 |
| Mouse anti-HNF4A | Abcam | ab41898 | 1:1,000 |
| Donkey anti-Goat IgG (H+L) Secondary Antibody, Alexa Fluor 647 | Life Technologies | A-21447 | 1:500 |
| Donkey anti-Mouse IgG (H+L) Secondary Antibody, Alexa Fluor 555 | Life Technologies | A-31570 | 1:500 |
| Donkey anti-Rabbit IgG (H+L) Secondary Antibody, Alexa Fluor 488 | Life Technologies | A-21206 | 1:500 |
| Flow cytometry | | | |
| Mouse anti-ASGR1-PE | SinoBiological | 10773-MM02-P | 5 μl/test |
| Mouse anti-CD14-APC | BD Biosciences | 561383 | 5 μl/test |
| Mouse anti-CD45-APC | BD Biosciences | 340943 | 5 μl/test |
| Mouse anti-CD86-PE | Biolegend | 374205 | 5 μl/test |
| Mouse anti-CD192(CCR2)-APC | Biolegend | 357207 | 5 μl/test |
| Western blotting | | | |
| Rabbit anti-AKT | Cell Signaling | 9272s | 1:1,000 |
| Rabbit anti-Phospho-AKT (Ser473) | Cell Signaling | 4060s | 1:2,000 |
| Rabbit anti-Phospho-AKT (Thr308) | Cell Signaling | 4056s | 1:1,000 |
| Rabbit anti-β-Actin (ACTB) | Cell Signaling | 4970S | 1:5,000 |
| Mouse anti-GAPDH | Santa Cruz Biotechnology | sc-47724 | 1:2,000 |
| Rabbit anti-Glycogen Synthase | Cell Signaling | 3886S | 1:1,000 |
| Rabbit anti-Phospho-Glycogen Synthase (Ser641) | Cell Signaling | 47043T | 1:1,000 |
| Rabbit anti-GSK3B | Cell Signaling | 12456T | 1:1,000 |
| Rabbit anti-Phospho-GSK3B (S9) | Cell Signaling | 5558T | 1:1,000 |
| Rabbit anti-Insulin receptor (IR) β | Cell Signaling | 23413t | 1:1,000 |
| Mouse anti-Phospho-Y | Cell Signaling | 96215S | 1:1,000 |
| Rabbit anti-IRS-1 | Cell Signaling | 2382S | 1:1,000 |
| Rabbit anti-Phospho-IRS-1 (Ser307) | Cell Signaling | 2381T | 1:1,000 |
| Rabbit anti-JNK | Cell Signaling | 9252s | 1:2,000 |
| Rabbit anti-Phospho-JNK (T183/Y185) | Cell Signaling | 4668t | 1:2,000 |
| Rabbit anti-PYGL | Proteintech | 15851-1-AP | 1:2,000 |
| Rabbit anti-Phospho-PYGL (Ser15) | Abcam | ab227043 | 1:2,000 |
| Rabbit anti p70 S6 Kinase (S6K) | Cell Signaling | 2708S | 1:1,000 |
| Rabbit anti-Phospho-p70 S6 Kinase (S6K) (T389) | Cell Signaling | 9205S | 1:1,000 |
| Peroxidase AffiniPure Goat Anti-Mouse IgG | Jackson ImmunoResearch | 115-035-062 | 1:10,000 |
| Peroxidase AffiniPure Goat Anti-Rabbit IgG | Jackson ImmunoResearch | 111-035-144 | 1:10,000 |
| Cytokine neutralization | | | |
| Infliximab | Selleckchem | A2019 | 5 μg/ml |
| Human IL-1 beta/IL-1F2 Antibody | R&D Systems | MAB201-SP | 0.2 μg/ml |

**Supplementary Table 3 | R software information and packages.**

| Package | Version | Software |
|---|---|---|
| annotate | 1.68.0 | Bioconductor |
| AnnotationDbi | 1.52.0 | Bioconductor |
| AnnotationForge | 1.32.0 | Bioconductor |
| ash | 1.0-15 | CRAN (R 4.0.2) |
| askpass | 1.1 | CRAN (R 4.0.2) |
| assertthat | 0.2.1 | CRAN (R 4.0.2) |
| beeswarm | 0.4.0 | CRAN (R 4.0.2) |
| Biobase | 2.50.0 | Bioconductor |
| BiocFileCache | 1.14.0 | Bioconductor |
| BiocGenerics | 0.36.1 | Bioconductor |
| BiocManager | 1.30.16 | CRAN (R 4.0.2) |
| BiocParallel | 1.24.1 | Bioconductor |
| biomaRt | 2.46.3 | Bioconductor |
| Biostrings | 2.58.0 | Bioconductor |
| bit | 4.0.4 | CRAN (R 4.0.2) |
| bit64 | 4.0.5 | CRAN (R 4.0.2) |
| bitops | 1.0-7 | CRAN (R 4.0.2) |
| blob | 1.2.2 | CRAN (R 4.0.2) |
| brio | 1.1.3 | CRAN (R 4.0.2) |
| cachem | 1.0.6 | CRAN (R 4.0.2) |
| calibrate | 1.7.7 | CRAN (R 4.0.2) |
| callr | 3.7.0 | CRAN (R 4.0.2) |
| Category | 2.56.0 | Bioconductor |
| caTools | 1.18.2 | CRAN (R 4.0.2) |
| cli | 3.2.0 | CRAN (R 4.0.2) |
| cluster | 2.1.2 | CRAN (R 4.0.2) |
| clusterProfiler | 3.18.1 | Bioconductor |
| colorspace | 2.0-3 | CRAN (R 4.0.2) |
| corrplot | 0.92 | CRAN (R 4.0.2) |
| cowplot | 1.1.1 | CRAN (R 4.0.2) |
| crayon | 1.5.0 | CRAN (R 4.0.2) |
| curl | 4.3.2 | CRAN (R 4.0.2) |
| data.table | 1.14.2 | CRAN (R 4.0.2) |
| DBI | 1.1.2 | CRAN (R 4.0.2) |
| dbplyr | 2.1.1 | CRAN (R 4.0.2) |
| DelayedArray | 0.16.3 | Bioconductor |
| desc | 1.4.1 | CRAN (R 4.0.2) |
| DESeq2 | 1.30.1 | Bioconductor |
| devtools | 2.4.3 | CRAN (R 4.0.2) |
| digest | 0.6.29 | CRAN (R 4.0.2) |
| DO.db | 2.9 | Bioconductor |
| DOSE | 3.16.0 | Bioconductor |
| downloader | 0.4 | CRAN (R 4.0.2) |
| dplyr | 1.0.8 | CRAN (R 4.0.2) |
| ellipsis | 0.3.2 | CRAN (R 4.0.2) |
| EnhancedVolcano | 1.8.0 | Bioconductor |

| | | |
|---|---|---|
| enrichplot | 1.10.2 | Bioconductor |
| extrafont | 0.17 | CRAN (R 4.0.2) |
| extrafontdb | 1 | CRAN (R 4.0.2) |
| fansi | 1.0.2 | CRAN (R 4.0.2) |
| farver | 2.1.0 | CRAN (R 4.0.2) |
| fastmap | 1.1.0 | CRAN (R 4.0.2) |
| fastmatch | 1.1-3 | CRAN (R 4.0.2) |
| fgsea | 1.16.0 | Bioconductor |
| fs | 1.5.2 | CRAN (R 4.0.2) |
| gage | 2.40.2 | Bioconductor |
| gageData | 2.28.0 | Bioconductor |
| genefilter | 1.72.1 | Bioconductor |
| geneplotter | 1.68.0 | Bioconductor |
| generics | 0.1.2 | CRAN (R 4.0.2) |
| GenomeInfoDb | 1.26.7 | Bioconductor |
| GenomeInfoDbData | 1.2.4 | Bioconductor |
| GenomicRanges | 1.42.0 | Bioconductor |
| ggalt | 0.4.0 | CRAN (R 4.0.2) |
| ggbeeswarm | 0.6.0 | CRAN (R 4.0.2) |
| ggforce | 0.3.3 | CRAN (R 4.0.2) |
| ggfun | 0.0.5 | CRAN (R 4.0.2) |
| ggplot2 | 3.3.5 | CRAN (R 4.0.2) |
| ggraph | 2.0.5 | CRAN (R 4.0.2) |
| ggrastr | 1.0.1 | CRAN (R 4.0.2) |
| ggrepel | 0.9.1 | CRAN (R 4.0.2) |
| glue | 1.6.2 | CRAN (R 4.0.2) |
| GO.db | 3.12.1 | Bioconductor |
| GOSemSim | 2.16.1 | Bioconductor |
| GOstats | 2.56.0 | Bioconductor |
| gplots | 3.1.1 | CRAN (R 4.0.2) |
| graph | 1.68.0 | Bioconductor |
| graphlayouts | 0.8.0 | CRAN (R 4.0.2) |
| gridExtra | 2.3 | CRAN (R 4.0.2) |
| GSEABase | 1.52.1 | Bioconductor |
| gtable | 0.3.0 | CRAN (R 4.0.2) |
| gtools | 3.9.2 | CRAN (R 4.0.2) |
| hms | 1.1.1 | CRAN (R 4.0.2) |
| httr | 1.4.2 | CRAN (R 4.0.2) |
| igraph | 1.2.11 | CRAN (R 4.0.2) |
| IRanges | 2.24.1 | Bioconductor |
| KEGGgraph | 1.50.0 | Bioconductor |
| KEGGREST | 1.30.1 | Bioconductor |
| KernSmooth | 2.23-20 | CRAN (R 4.0.2) |
| labeling | 0.4.2 | CRAN (R 4.0.2) |
| lattice | 0.20-45 | CRAN (R 4.0.2) |
| lifecycle | 1.0.1 | CRAN (R 4.0.2) |
| limma | 3.46.0 | Bioconductor |
| locfit | 1.5-9.4 | CRAN (R 4.0.2) |
| magrittr | 2.0.2 | CRAN (R 4.0.2) |

| | | |
|---|---|---|
| maps | 3.4.0 | CRAN (R 4.0.2) |
| marray | 1.68.0 | Bioconductor |
| MASS | 7.3-55 | CRAN (R 4.0.2) |
| Matrix | 1.4-0 | CRAN (R 4.0.2) |
| MatrixGenerics | 1.2.1 | Bioconductor |
| matrixStats | 0.61.0 | Bioconductor |
| memoise | 2.0.1 | CRAN (R 4.0.2) |
| munsell | 0.5.0 | CRAN (R 4.0.2) |
| openssl | 2.0.0 | CRAN (R 4.0.2) |
| org.Hs.eg.db | 3.12.0 | Bioconductor |
| pacman | 0.5.1 | CRAN (R 4.0.2) |
| pathview | 1.30.1 | Bioconductor |
| pheatmap | 1.0.12 | CRAN (R 4.0.2) |
| pillar | 1.7.0 | CRAN (R 4.0.2) |
| pkgbuild | 1.3.1 | CRAN (R 4.0.2) |
| pkgconfig | 2.0.3 | CRAN (R 4.0.2) |
| pkgload | 1.2.4 | CRAN (R 4.0.2) |
| plyr | 1.8.6 | CRAN (R 4.0.2) |
| png | 0.1-7 | CRAN (R 4.0.2) |
| polyclip | 1.10-0 | CRAN (R 4.0.2) |
| prettyunits | 1.1.1 | CRAN (R 4.0.2) |
| processx | 3.5.2 | CRAN (R 4.0.2) |
| progress | 1.2.2 | CRAN (R 4.0.2) |
| proj4 | 1.0-11 | CRAN (R 4.0.2) |
| ps | 1.6.0 | CRAN (R 4.0.2) |
| purrr | 0.3.4 | CRAN (R 4.0.2) |
| qvalue | 2.22.0 | Bioconductor |
| R.methodsS3 | 1.8.1 | CRAN (R 4.0.2) |
| R.oo | 1.24.0 | CRAN (R 4.0.2) |
| R.utils | 2.11.0 | CRAN (R 4.0.2) |
| R6 | 2.5.1 | CRAN (R 4.0.2) |
| ragg | 1.2.2 | CRAN (R 4.0.2) |
| rappdirs | 0.3.3 | CRAN (R 4.0.2) |
| RBGL | 1.66.0 | Bioconductor |
| RColorBrewer | 1.1-2 | CRAN (R 4.0.2) |
| Rcpp | 1.0.8.3 | CRAN (R 4.0.2) |
| RCurl | 1.98-1.6 | CRAN (R 4.0.2) |
| RDAVIDWebService | 1.28.0 | Bioconductor |
| remotes | 2.4.2 | CRAN (R 4.0.2) |
| reshape2 | 1.4.4 | CRAN (R 4.0.2) |
| Rgraphviz | 2.34.0 | Bioconductor |
| rJava | 1.0-6 | CRAN (R 4.0.2) |
| rlang | 1.0.2 | CRAN (R 4.0.2) |
| rprojroot | 2.0.2 | CRAN (R 4.0.2) |
| RSQLite | 2.2.10 | CRAN (R 4.0.2) |
| rstudioapi | 0.13 | CRAN (R 4.0.2) |
| Rtsne | 0.15 | CRAN (R 4.0.2) |
| Rttf2pt1 | 1.3.10 | CRAN (R 4.0.2) |
| rvcheck | 0.2.1 | CRAN (R 4.0.2) |

| | | |
|---|---|---|
| S4Vectors | 0.28.1 | Bioconductor |
| scales | 1.1.1 | CRAN (R 4.0.2) |
| scatterpie | 0.1.7 | CRAN (R 4.0.2) |
| sessioninfo | 1.2.2 | CRAN (R 4.0.2) |
| shadowtext | 0.1.1 | CRAN (R 4.0.2) |
| stringi | 1.7.6 | CRAN (R 4.0.2) |
| stringr | 1.4.0 | CRAN (R 4.0.2) |
| SummarizedExperiment | 1.20.0 | Bioconductor |
| survival | 3.3-1 | CRAN (R 4.0.2) |
| svglite | 2.1.0 | CRAN (R 4.0.2) |
| systemfonts | 1.0.4 | CRAN (R 4.0.2) |
| testthat | 3.1.2 | CRAN (R 4.0.2) |
| textshaping | 0.3.6 | CRAN (R 4.0.2) |
| tibble | 3.1.6 | CRAN (R 4.0.2) |
| tidygraph | 1.2.0 | CRAN (R 4.0.2) |
| tidyr | 1.2.0 | CRAN (R 4.0.2) |
| tidyselect | 1.1.2 | CRAN (R 4.0.2) |
| tweenr | 1.0.2 | CRAN (R 4.0.2) |
| tximport | 1.18.0 | Bioconductor |
| usethis | 2.1.5 | CRAN (R 4.0.2) |
| utf8 | 1.2.2 | CRAN (R 4.0.2) |
| vctrs | 0.3.8 | CRAN (R 4.0.2) |
| vipor | 0.4.5 | CRAN (R 4.0.2) |
| viridis | 0.6.2 | CRAN (R 4.0.2) |
| viridisLite | 0.4.0 | CRAN (R 4.0.2) |
| withr | 2.5.0 | CRAN (R 4.0.2) |
| XML | 3.99-0.9 | CRAN (R 4.0.2) |
| xml2 | 1.3.3 | CRAN (R 4.0.2) |
| xtable | 1.8-4 | CRAN (R 4.0.2) |
| XVector | 0.30.0 | Bioconductor |
| yulab.utils | 0.0.4 | CRAN (R 4.0.2) |
| zlibbioc | 1.36.0 | Bioconductor |

**Supplementary Table 4 | *P* values of statistically significant results.**

| Figure | Panel | Condition | *P* value |
|---|---|---|---|
| Main Figures | | | |
| 1a | | I vs. G (60 min)<br>I vs. G (120 min)<br>I vs. G (180 min) | 0.0004<br><0.0001<br>0.0208 |
| 1c | *PCK1* | C vs. G<br>I vs. G<br>G vs. G+I | 0.0076<br>0.0027<br>0.0255 |
| | *G6PC* | C vs. G<br>C vs. G+I<br>I vs. G<br>G vs. G+I | <0.0001<br>0.0282<br><0.0001<br>0.0003 |
| | *GCK* | C vs. I<br>C vs. G+I<br>I vs. G<br>G vs. G+I | 0.0480<br>0.0248<br>0.0170<br>0.0091 |
| 1d | pAKT(T308)/AKT | 10 min<br>20 min<br>30 min | 0.0454<br>0.0327<br>0.0051 |
| | pAKT(S473)/AKT | 20 min<br>30 min | 0.0350<br>0.0295 |
| | pS6K1(T389)/S6K1 | 15 min<br>30 min | 0.0075<br>0.0210 |
| | pPYGL(S15)/PYGL | 30 min | 0.0355 |
| 1e | | G vs. I | 0.0239 |
| 2b | LDH | M1 vs. M0<br>M1 vs. C | 0.0203<br>0.0052 |
| | Cl. caspase 3 | M1 vs. M0<br>M1 vs. C | 0.0007<br>0.0004 |
| 2c | WTC<br>pJNK(T183/Y185) | M1 vs. M0<br>M1 vs. C | 0.0010<br>0.0005 |
| | CW10030<br>pJNK(T183/Y185) | M1 vs. M0<br>M1 vs. C | 0.0245<br>0.0035 |
| 2d | WTC IL1β | M1 vs. M0<br>M1 vs. C | 0.0005<br>0.0001 |
| | WTC TNFα | M1 vs. M0<br>M1 vs. C | 0.0049<br>0.0046 |
| | CW10030 IL1β | M1 vs. M0<br>M1 vs. C | <0.0001<br><0.0001 |
| | CW10030 TNFα | M1 vs. M0<br>M1 vs. C | 0.0026<br>0.0021 |
| 2e | | M1 v. M0 (30 min)<br>M1 vs. C (30 min)<br>M1 v. M0 (60 min)<br>M1 vs. C (60 min)<br>M1 v. M0 (120 min)<br>M1 vs. C (120 min) | 0.0034<br><0.0001<br>0.0037<br><0.0001<br>0.0050<br><0.0001 |
| 2f | pAKT(T308)/AKT | M1 vs. M0 | 0.0009 |

| | | | |
|---|---|---|---|
| | | M1 vs. C | 0.0134 |
| | pAKT(S473)/AKT | M1 vs. M0<br>M1 vs. C | 0.0017<br>0.0148 |
| | pPYGL(S15)/PYGL | M1 vs. M0<br>M1 vs. C | 0.0122<br>0.0475 |
| 2g | *G6PC* | M1 vs. M0<br>M1 vs. C | 0.0440<br>0.0281 |
| | *PCK1* | M1 vs. M0<br>M1 vs. C | 0.0009<br>0.0009 |
| | *GCK* | M1 vs. M0<br>M1 vs. C | 0.0136<br>0.0008 |
| 2h | | C vs. N | 0.0006 |
| 4b | | M1 vs. M1+anti-TNFα<br>M1 vs. M1+anti-TNFα+anti-IL1β | 0.0024<br>0.0003 |
| 4c | | M1 vs. M1+anti-IL1β<br>M1 vs. M1+anti-TNFα+anti-IL1β | 0.0187<br>0.0282 |
| 4d | | M1 vs. M1+anti-IL1β (30 min)<br>M1 vs. M1+anti-TNFα+anti-IL1β (30 min)<br>M1 vs. M1+anti-IL1β (60 min)<br>M1 vs. M1+anti-TNFα+anti-IL1β (60 min)<br>M1 vs. M1+anti-TNFα (120 min)<br>M1 vs. M1+anti-IL1β (120 min)<br>M1 vs. M1+anti-TNFα+anti-IL1β (120 min) | 0.0039<br>0.0002<br>0.0009<br>0.0027<br>0.0017<br>0.0179<br><0.0001 |
| 4e | pAKT(T308)/AKT | M1 vs. M1+anti-TNFα+anti-IL1β | 0.0073 |
| | pAKT(S473)/AKT | M1 vs. M1+anti-TNFα+anti-IL1β | 0.0273 |
| 4f | *PCK1* | M1 vs. M1+anti-TNFα+anti-IL1β | 0.0284 |
| | *G6PC* | M1 vs. M1+anti-TNFα<br>M1 vs. M1+anti-TNFα+anti-IL1β | 0.0070<br>0.0125 |
| | *GCK* | M1 vs. M1+anti-TNFα<br>M1 vs. M1+anti-IL1β<br>M1 vs. M1+anti-TNFα+anti-IL1β | 0.0361<br>0.0037<br>0.0055 |
| 4g | pPYGL(S15)/PYGL | M1 vs. M1+anti-TNFα+anti-IL1β | 0.0091 |
| 5a | IL1β | M1 vs. M0<br>M1 vs. C | 0.0261<br>0.0069 |
| | TNFα | M1 vs. M0<br>M1 vs. C | <0.0001<br><0.0001 |
| 5c | *JUN* | M1 vs. M0<br>M1 vs. C<br>M1 vs. M1+NAbs | <0.0001<br><0.0001<br>0.0142 |
| | *CASP1* | M1 vs. M0<br>M1 vs. C | <0.0001<br><0.0001 |
| | *NFKB2* | M1 vs. M0<br>M1 vs. C<br>M1 vs. M1+NAbs | 0.0001<br>0.0002<br>0.0005 |
| | *TNF* | M1 vs. M0<br>M1 vs. C<br>M1 vs. M1+NAbs | <0.0001<br><0.0001<br>0.0235 |
| 5d | Primary cells | M1 vs. C<br>M1 vs. M1+NAbs | 0.0279<br>0.0020 |

| | | | |
|---|---|---|---|
| | CW10030 iPSC-derived cells | M1 vs. C<br>M1 vs. M1+NAbs | 0.0016<br>0.0066 |
| 5e | Primary cells pAKT(S473)/AKT | M1 vs. C<br>M1 vs. M1+NAbs | 0.0063<br>0.0020 |
| | CW10030 iPSC-derived cells pAKT(S473)/AKT | M1 vs. C<br>M1 vs. M1+NAbs | 0.0435<br>0.0085 |
| 5f | Primary cells *PCK1* | M1 vs. C<br>M1 vs. M1+NAbs | 0.0042<br>0.0057 |
| | Primary cells *GCK* | M1 vs. C<br>M1 vs. M1+NAbs | 0.0458<br>0.0164 |
| | CW10030 iPSC-derived cells *PCK1* | M1 vs. C<br>M1 vs. M1+NAbs | 0.0389<br>0.0096 |
| | CW10030 iPSC-derived cells *GCK* | M1 vs. C<br>M1 vs. M1+NAbs | 0.0306<br>0.0232 |
| Supplementary Figures | | | |
| 2a | | C vs. I (30 min) | 0.0336 |
| 2b | *INSR* | C vs. I<br>C vs. G<br>C vs. G+I<br>I vs. G+I<br>G vs. G+I | 0.0010<br>0.0008<br><0.0001<br>0.0338<br>0.0459 |
| | *IRS1* | C vs. G<br>C vs. G+I<br>I vs. G+I | 0.0119<br>0.0015<br>0.0265 |
| | *IRS2* | C vs. I<br>C vs. G<br>C vs. G+I<br>I vs. G+I<br>G vs. G+I | 0.0015<br>0.0025<br><0.0001<br>0.0032<br>0.0019 |
| 2c | pY/INSR | 5 min<br>15 min<br>30 min | 0.0006<br>0.0005<br>0.0002 |
| 3d | *CASP1* | M1 vs. M0<br>M1 vs. C | <0.0001<br><0.0001 |
| | *CASP4* | M1 vs. M0<br>M1 vs. C | <0.0001<br><0.0001 |
| | *CASP5* | M1 vs. M0<br>M1 vs. C | <0.0001<br><0.0001 |
| 3e | WTC *NFKB2* | M1 vs. M0<br>M1 vs. C | <0.0001<br><0.0001 |
| | WTC *TNF* | M1 vs. M0<br>M1 vs. C | <0.0001<br><0.0001 |
| | CW10030 *NFKB2* | M1 vs. M0<br>M1 vs. C | 0.0176<br>0.0055 |
| | CW10030 *TNF* | M1 vs. M0<br>M1 vs. C | <0.0001<br><0.0001 |
| 4d | *IRS1* | M1 vs. M0<br>M1 vs. C | 0.0017<br>0.0039 |
| | *IRS2* | M1 vs. M0 | 0.0028 |

| | | | |
|---|---|---|---|
| | | M1 vs. C | 0.0005 |
| 4e | | M1 vs. M0<br>M1 vs. C | 0.0340<br>0.0014 |
| 4f | | M1 vs. M0<br>M1 vs. C | 0.0416<br>0.0072 |
| 4h | | M1 vs. M0<br>M1 vs. C | 0.0411<br>0.0349 |
| 4j | | L vs. C | 0.0005 |
| 6a | *TNF* | M1 vs. M1+anti-TNFα<br>M1 vs. M1+anti-TNFα+anti-IL1β | 0.0309<br>0.0002 |
| 6c | *IRS1* | M1 vs. M1+anti-TNFα<br>M1 vs. M1+anti-IL1β | 0.0315<br>0.0199 |
| | *IRS2* | M1 vs. M1+anti-TNFα<br>M1 vs. M1+anti-IL1β<br>M1 vs. M1+anti-TNFα+anti-IL1β | <0.0001<br><0.0001<br><0.0001 |
| 7a | | C 2h vs. M1 2h | 0.0151 |
| 7b | | M1 vs. C<br>M1 vs. M1+NAbs | 0.0064<br>0.0067 |
| 7c | CW10030 iPSC-derived cells pPYGL(S15)/PYGL | M1 vs. C<br>M1 vs. M1+NAbs | 0.0304<br>0.0026 |
| 7d | | M1 vs. C | 0.0376 |
