# Supplementary material for: Modeling and therapeutic targeting of inflammation-induced hepatic insulin resistance using human iPSC-derived hepatocytes and macrophages
Source: Nat Commun. 2023 Jul 3;14:3902. doi: 10.1038/s41467-023-39311-w (PMC10318012; doi:10.1038/s41467-023-39311-w)
Supplement: Supplementary file 3 — Description of Additional Supplementary Files [file 41467_2023_39311_MOESM3_ESM.pdf]

Description of Additional Supplementary Files

File Name: Supplementary Data 1
Description: Processed RNAseq data
