# Supplementary material for: Modeling and therapeutic targeting of inflammation-induced hepatic insulin resistance using human iPSC-derived hepatocytes and macrophages
Source: Nat Commun. 2023 Jul 3;14:3902. doi: 10.1038/s41467-023-39311-w (PMC10318012; doi:10.1038/s41467-023-39311-w)
Supplement: Supplementary file 5 — Reporting Summary [file 41467_2023_39311_MOESM5_ESM.pdf]

nature portfolio

Corresponding author(s): Holger Willenbring

Last updated by author(s): May 21, 2023

Reporting Summary

Nature Portfolio wishes to improve the reproducibility of the work that we publish. This form provides structure for consistency and transparency in reporting. For further information on Nature Portfolio policies, see our Editorial Policies and the Editorial Policy Checklist.

Statistics

For all statistical analyses, confirm that the following items are present in the figure legend, table legend, main text, or Methods section.

n/a	Confirmed	
☐	☒	The exact sample size (*n*) for each experimental group/condition, given as a discrete number and unit of measurement
☐	☒	A statement on whether measurements were taken from distinct samples or whether the same sample was measured repeatedly
☐	☒	The statistical test(s) used AND whether they are one- or two-sided<br>*Only common tests should be described solely by name; describe more complex techniques in the Methods section.*
☒	☐	A description of all covariates tested
☐	☒	A description of any assumptions or corrections, such as tests of normality and adjustment for multiple comparisons
☐	☒	A full description of the statistical parameters including central tendency (e.g. means) or other basic estimates (e.g. regression coefficient) AND variation (e.g. standard deviation) or associated estimates of uncertainty (e.g. confidence intervals)
☐	☒	For null hypothesis testing, the test statistic (e.g. *F*, *t*, *r*) with confidence intervals, effect sizes, degrees of freedom and *P* value noted<br>*Give P values as exact values whenever suitable.*
☒	☐	For Bayesian analysis, information on the choice of priors and Markov chain Monte Carlo settings
☒	☐	For hierarchical and complex designs, identification of the appropriate level for tests and full reporting of outcomes
☒	☐	Estimates of effect sizes (e.g. Cohen's *d*, Pearson's *r*), indicating how they were calculated

Our web collection on statistics for biologists contains articles on many of the points above.

Software and code

Policy information about availability of computer code

Data collection	No code was used for data collection.
Data analysis	GraphPad Prism (v9.5.1.773), LEGENDplex data analysis software (v8.0), FloJo (v10.6.1), Quantstudio Real-Time PCR software (v1.3), FIJI (v1.53q), STAR (v2.7.2b), DESeq2 (v1.30.1), Ingenuity Pathway analysis (IPA) (v21.0.1), R software (v4.0.2), Venny (v2.1.0; (https://bioinfogp.cnb.csic.es/tools/venny/index.html)), annotate (v1.68.0; Bioconductor), AnnotationDbi (v1.52.0; Bioconductor), AnnotationForge (v1.32.0; Bioconductor), ash (v1.0-15; CRAN (R 4.0.2)), askpass (v1.1; CRAN (R 4.0.2)), assertthat (v0.2.1; CRAN (R 4.0.2)), beeswarm (v0.4.0; CRAN (R 4.0.2)), Biobase (v2.50.0; Bioconductor), BiocFileCache (v1.14.0; Bioconductor), BiocGenerics (v0.36.1; Bioconductor), BiocManager v1.30.16; CRAN (R 4.0.2)), BiocParallel (v1.24.1; Bioconductor), biomaRt (v2.46.3; Bioconductor), Biostrings (v2.58.0; Bioconductor), bit (v4.0.4; CRAN (R 4.0.2)), bit64 (v4.0.5; CRAN (R 4.0.2)), bitops (v1.0-7; CRAN (R 4.0.2)), blob (v1.2.2; CRAN (R 4.0.2)), brio (v1.1.3; CRAN (R 4.0.2)), cachem (v1.0.6; CRAN (R 4.0.2)), calibrate (v1.7.7; CRAN (R 4.0.2)), callr (v3.7.0; CRAN (R 4.0.2)), Category (v2.56.0; Bioconductor), caTools (v1.18.2; CRAN (R 4.0.2)), cli (v3.2.0; CRAN (R 4.0.2)), cluster (v2.1.2; CRAN (R 4.0.2)), clusterProfiler (v3.18.1; Bioconductor), colorspace (v2.0-3; CRAN (R 4.0.2)), corrplot (v0.92; CRAN (R 4.0.2)), cowplot (v1.1.1; CRAN (R 4.0.2)), crayon (v1.5.0; CRAN (R 4.0.2)), curl (v4.3.2; CRAN (R 4.0.2)), data.table (v1.14.2; CRAN (R 4.0.2), DBI (v1.1.2; CRAN (R 4.0.2)), dbplyr (v2.1.1; CRAN (R 4.0.2)), DelayedArray (v0.16.3; Bioconductor), desc (v1.4.1; CRAN (R 4.0.2)), devtools (v2.4.3; CRAN (R 4.0.2)), digest (v0.6.29; CRAN (R 4.0.2)), DO.db (v2.9; Bioconductor), DOSE (v3.16.0; Bioconductor), downloader (v0.4; CRAN (R 4.0.2)), dplyr (v1.0.8; CRAN (R 4.0.2)), ellipsis (v0.3.2; CRAN (R 4.0.2)), EnhancedVolcano (v1.8.0; Bioconductor), enrichplot (v1.10.2; Bioconductor), extrafont (v0.17; CRAN (R 4.0.2)), extrafontdb; (v1 CRAN (R 4.0.2)), fansi (v1.0.2; CRAN (R 4.0.2)), farver (v2.1.0; CRAN (R 4.0.2)), fastmap (v1.1.0; CRAN (R 4.0.2)), fastmatch (v1.1-3; CRAN (R 4.0.2)), fgsea (v1.16.0; Bioconductor), fs (v1.5.2; CRAN (R 4.0.2)), gage (v2.40.2; Bioconductor), gageData (v2.28.0; Bioconductor), genefilter (v1.72.1; Bioconductor), geneplotter (v1.68.0; Bioconductor), generics (v0.1.2; CRAN (R 4.0.2)), GenomeInfoDb (v1.26.7; Bioconductor), GenomeInfoDbData (v1.2.4; Bioconductor), GenomicRanges (v1.42.0; Bioconductor), ggalt (v0.4.0; CRAN (R 4.0.2)), ggbeeswarm (v0.6.0; CRAN (R 4.0.2)), ggforce (v0.3.3; CRAN (R 4.0.2)), ggfun (v0.0.5; CRAN (R 4.0.2)), ggplot2 (v3.3.5; CRAN (R 4.0.2)), ggraph (v2.0.5; CRAN (R

4.0.2)), ggrastr (v1.0.1; CRAN (R 4.0.2)), ggrepel (v0.9.1; CRAN (R 4.0.2)), glue (v1.6.2; CRAN (R 4.0.2)), GO.db (v3.12.1; Bioconductor), GOSemSim (v2.16.1; Bioconductor), GOstats (v2.56.0; Bioconductor), gplots (v3.1.1; CRAN (R 4.0.2)), graph (v1.68.0; Bioconductor), graphlayouts (v0.8.0; CRAN (R 4.0.2)), gridExtra (v2.3; CRAN (R 4.0.2)), GSEABase (v1.52.1; Bioconductor), gtable (v0.3.0; CRAN (R 4.0.2)), gtools (v3.9.2; CRAN (R 4.0.2)), hms (v1.1.1; CRAN (R 4.0.2)), httr (v1.4.2; CRAN (R 4.0.2)), igraph (v1.2.11; CRAN (R 4.0.2)), IRanges (v2.24.1; Bioconductor), KEGGgraph (v1.50.0; Bioconductor), KEGGREST (v1.30.1; Bioconductor), KernSmooth (v2.23-20; CRAN (R 4.0.2)), labeling (v0.4.2; CRAN (R 4.0.2)), lattice (v0.20-45; CRAN (R 4.0.2)), lifecycle (v1.0.1; CRAN (R 4.0.2)), limma (v3.46.0; Bioconductor), locfit (v1.5-9.4; CRAN (R 4.0.2)), magrittr (v2.0.2; CRAN (R 4.0.2)), maps (v3.4.0; CRAN (R 4.0.2)), marray (v1.68.0; Bioconductor), MASS (v7.3-55; CRAN (R 4.0.2)), Matrix (v1.4-0; CRAN (R 4.0.2)), MatrixGenerics (v1.2.1; Bioconductor), matrixStats (v0.61.0; Bioconductor), memoise (v2.0.1; CRAN (R 4.0.2)), munsell (v0.5.0; CRAN (R 4.0.2)), openssl (v2.0.0; CRAN (R 4.0.2)), org.Hs.eg.db (v3.12.0; Bioconductor), pacman (v0.5.1; CRAN (R 4.0.2)), pathview (v1.30.1; Bioconductor), pheatmap (v1.0.12; CRAN (R 4.0.2)), pillar (v1.7.0; CRAN (R 4.0.2)), pkgbuild (v1.3.1; CRAN (R 4.0.2)), pkgconfig (v2.0.3; CRAN (R 4.0.2)), pkgload (v1.2.4; CRAN (R 4.0.2)), plyr (v1.8.6; CRAN (R 4.0.2)), png (v0.1-7; CRAN (R 4.0.2)), polyclip (v1.10-0; CRAN (R 4.0.2)), prettyunits (v1.1.1; CRAN (R 4.0.2)), processx (v3.5.2; CRAN (R 4.0.2)), progress (v1.2.2; CRAN (R 4.0.2)), proj4 (v1.0-11; CRAN (R 4.0.2)), ps (v1.6.0; CRAN (R 4.0.2)), purrr (v0.3.4; CRAN (R 4.0.2)), qvalue (v2.22.0; Bioconductor), R.methodsS3 (v1.8.1; CRAN (R 4.0.2)), R.oo (v1.24.0; CRAN (R 4.0.2)), R.utils (v2.11.0; CRAN (R 4.0.2)), R6 (v2.5.1; CRAN (R 4.0.2)), ragg (v1.2.2; CRAN (R 4.0.2)), rappdirs (v0.3.3; CRAN (R 4.0.2)), RBGL (v1.66.0; Bioconductor), RColorBrewer (v1.1-2; CRAN (R 4.0.2)), Rcpp (v1.0.8.3; CRAN (R 4.0.2)), RCurl (v1.98-1.6; CRAN (R 4.0.2)), RDAVIDWebService (v1.28.0; Bioconductor), remotes (v2.4.2; CRAN (R 4.0.2)), reshape2 (v1.4.4; CRAN (R 4.0.2)), Rgraphviz (v2.34.0; Bioconductor), rJava (v1.0-6; CRAN (R 4.0.2)), rlang (v1.0.2; CRAN (R 4.0.2)), rprojroot (v2.0.2; CRAN (R 4.0.2)), RSQLite (v2.2.10; CRAN (R 4.0.2)), rstudioapi (v0.13; CRAN (R 4.0.2)), Rtsne (v0.15; CRAN (R 4.0.2)), Rttf2pt1 (v1.3.10; CRAN (R 4.0.2)), rvcheck (v0.2.1; CRAN (R 4.0.2)), S4Vectors (v0.28.1; Bioconductor), scales (v1.1.1; CRAN (R 4.0.2)), scatterpie (v0.1.7; CRAN (R 4.0.2)), sessioninfo (v1.2.2; CRAN (R 4.0.2)), shadowtext (v0.1.1; CRAN (R 4.0.2)), stringi (v1.7.6; CRAN (R 4.0.2)), stringr (v1.4.0; CRAN (R 4.0.2)), SummarizedExperiment (v1.20.0; Bioconductor), survival (v3.3-1; CRAN (R 4.0.2)), svglite (v2.1.0; CRAN (R 4.0.2)), systemfonts (v1.0.4; CRAN (R 4.0.2)), testthat (v3.1.2; CRAN (R 4.0.2)), textshaping (v0.3.6; CRAN (R 4.0.2)), tibble (v3.1.6; CRAN (R 4.0.2)), tidygraph (v1.2.0; CRAN (R 4.0.2)), tidyr (v1.2.0; CRAN (R 4.0.2)), tidyselect (v1.1.2; CRAN (R 4.0.2)), tweenr (v1.0.2; CRAN (R 4.0.2)), tximport (v1.18.0; Bioconductor), usethis (v2.1.5; CRAN (R 4.0.2)), utf8 (v1.2.2; CRAN (R 4.0.2)), vctrs (v0.3.8; CRAN (R 4.0.2)), vipor (v0.4.5; CRAN (R 4.0.2)), viridis (v0.6.2; CRAN (R 4.0.2)), viridisLite (v0.4.0; CRAN (R 4.0.2)), withr (v2.5.0; CRAN (R 4.0.2)), XML (v3.99-0.9; CRAN (R 4.0.2)), xml2 (v1.3.3; CRAN (R 4.0.2)), xtable (v1.8-4; CRAN (R 4.0.2)), XVector (v0.30.0; Bioconductor), yulab.utils (v0.0.4; CRAN (R 4.0.2)), zlibbioc (v1.36.0; Bioconductor)

For manuscripts utilizing custom algorithms or software that are central to the research but not yet described in published literature, software must be made available to editors and reviewers. We strongly encourage code deposition in a community repository (e.g. GitHub). See the Nature Portfolio guidelines for submitting code & software for further information.

Data

Policy information about availability of data

All manuscripts must include a data availability statement. This statement should provide the following information, where applicable:

- Accession codes, unique identifiers, or web links for publicly available datasets
- A description of any restrictions on data availability
- For clinical datasets or third party data, please ensure that the statement adheres to our policy

Source data are provided in the Source Data file. Primer sequences, antibody sources and software information are provided in Supplementary Tables 1-3. Raw RNAseq data were deposited in the NCBI Gene Expression Omnibus database repository under accession number GSE228765. Processed RNAseq data are provided in Supplementary Data 1. RNAseq reads were aligned to the human reference genome GRCh38.96.

Human research participants

Policy information about studies involving human research participants and Sex and Gender in Research.

Reporting on sex and gender	*Use the terms sex (biological attribute) and gender (shaped by social and cultural circumstances) carefully in order to avoid confusing both terms. Indicate if findings apply to only one sex or gender; describe whether sex and gender were considered in study design whether sex and/or gender was determined based on self-reporting or assigned and methods used. Provide in the source data disaggregated sex and gender data where this information has been collected, and consent has been obtained for sharing of individual-level data; provide overall numbers in this Reporting Summary. Please state if this information has not been collected. Report sex- and gender-based analyses where performed, justify reasons for lack of sex- and gender-based analysis.*
Population characteristics	*Describe the covariate-relevant population characteristics of the human research participants (e.g. age, genotypic information, past and current diagnosis and treatment categories). If you filled out the behavioural & social sciences study design questions and have nothing to add here, write "See above."*
Recruitment	*Describe how participants were recruited. Outline any potential self-selection bias or other biases that may be present and how these are likely to impact results.*
Ethics oversight	*Identify the organization(s) that approved the study protocol.*

Note that full information on the approval of the study protocol must also be provided in the manuscript.

Field-specific reporting

Please select the one below that is the best fit for your research. If you are not sure, read the appropriate sections before making your selection.

☒ Life sciences ☐ Behavioural & social sciences ☐ Ecological, evolutionary & environmental sciences

For a reference copy of the document with all sections, see nature.com/documents/nr-reporting-summary-flat.pdf

Life sciences study design

All studies must disclose on these points even when the disclosure is negative.

Sample size	Sample size was chosen based on published examples minimizing assay-based errors, allowing for statistical analysis and ascertaining reproducibility of results: Molinaro et al. 2020 (DOI: 10.1038/s41598-020-68721-9); Titchenell et al. 2015 (DOI: 10.1038/ncomms8078).
Data exclusions	No data were excluded.
Replication	All experiments were repeated independently at least 3 times, except for Supplementary Fig. 1e (flow cytometry analysis of freshly thawed primary human hepatocytes). The number of biological replicates of each experiment is stated in the legends. Key findings generated with the human iPSC line GM25256 (WTC) were replicated using the human iPSC line CW10030 and primary human cells.
Randomization	The study includes no experiments dependent on randomized allocation of samples into experimental groups.
Blinding	The study includes no experiments dependent on group allocation and blinding.

Reporting for specific materials, systems and methods

We require information from authors about some types of materials, experimental systems and methods used in many studies. Here, indicate whether each material, system or method listed is relevant to your study. If you are not sure if a list item applies to your research, read the appropriate section before selecting a response.

Materials & experimental systems

n/a	Involved in the study	
☐	☒	Antibodies
☐	☒	Eukaryotic cell lines
☒	☐	Palaeontology and archaeology
☒	☐	Animals and other organisms
☒	☐	Clinical data
☒	☐	Dual use research of concern

Methods

n/a	Involved in the study	
☒	☐	ChIP-seq
☐	☒	Flow cytometry
☒	☐	MRI-based neuroimaging

Antibodies

Antibodies used

Antibody concentrations are provided in Supplementary Table 2.
Immunofluorescence:
Goat anti-Albumin; Bethyl Laboratories Cat#A80-229A
Rabbit anti-Cleaved Caspase 3(Asp175); Cell Signaling Cat#9661s
Mouse anti-HNF4A; Abcam Cat#ab41898
Donkey anti-Goat IgG (H+L) Secondary Antibody, Alexa Fluor 647; Life Technologies Cat#A-21447
Donkey anti-Mouse IgG (H+L) Secondary Antibody, Alexa Fluor 555; Life Technologies Cat#A-31570
Donkey anti-Rabbit IgG (H+L) Secondary Antibody, Alexa Fluor 488; Life Technologies Cat#A-21206
Flow cytometry:
Mouse anti-ASGR1-PE; SinoBiological Cat#10773-MM02-P
Mouse anti-CD14-APC; BD Biosciences Cat#561383
Mouse anti-CD45-APC; BD Biosciences Cat#340943
Mouse anti-CD86-PE; Biolegend Cat#374205
Mouse anti-CD192(CCR2)-APC; Biolegend Cat#357207
Western blotting:
Rabbit anti-AKT; Cell Signaling Cat#9272s
Rabbit anti-Phospho-AKT (Ser473); Cell Signaling Cat#4060s
Rabbit anti-Phospho-AKT (Thr308); Cell Signaling Cat#4056s
Rabbit anti-β-Actin (ACTB); Cell Signaling Cat#4970S
Mouse anti-GAPDH; Santa Cruz Cat#sc-47724
Rabbit anti-JNK; Cell Signaling Cat#9252s
Rabbit anti-Phospho-JNK; Cell Signaling Cat#4668t

	Rabbit anti-Glycogen Synthase; Cell Signaling Cat#3886S<br>Rabbit anti-Phospho-Glycogen Synthase (Ser641); Cell Signaling Cat#47043T<br>Rabbit anti-GSK3B; Cell Signaling 12456T<br>Rabbit anti-Phospho-GSK3B (S9); Cell Signaling 5558T<br>Rabbit anti-Insulin receptor (IR) β; Cell Signaling 23413t<br>Mouse anti-Phospho-Y; Cell Signaling 96215S<br>Rabbit anti-PYGL; Proteintech Cat#15851-1-AP<br>Rabbit anti-Phospho-PYGL (Ser15); Abcam Cat#ab227043<br>Rabbit anti-IRS-1; Cell Signaling Cat#2382S<br>Rabbit anti-Phospho-IRS-1 (Ser307); Cell Signaling Cat#2381T<br>Rabbit anti p70 S6 Kinase (S6K); Cell Signaling Cat#2708S<br>Rabbit anti-Phospho-p70 S6 Kinase (S6K) (T389); Cell Signaling Cat#9205S<br>Peroxidase AffiniPure Goat Anti-Mouse IgG; Jackson ImmunoResearch Cat#115-035-062<br>Peroxidase AffiniPure Goat Anti-Rabbit IgG; Jackson ImmunoResearch Cat#111-035-144<br>Cytokine neutralization:<br>Infliximab; Selleckchem Cat#A2019<br>Human IL-1 beta/IL-1F2 Antibody; R&D Systems Cat#MAB201-SP
Validation	Validation information can be found on the manufacturers' websites using the antibody Cat#:<br>https://www.abcam.com/primary-antibodies/how-we-validate-our-antibodies<br>https://www.bdbiosciences.com/en-us/products/reagents/flow-cytometry-reagents/research-reagents/quality-and-reproducibility<br>https://www.biolegend.com/en-us/quality/quality-control<br>https://www.cellsignal.com/about-us/cst-antibody-validation-principles<br>https://www.fortislife.com/antibody-validation<br>https://www.rndsystems.com/products/human-il-1beta-il-1f2-antibody-8516_mab201<br>https://www.scbt.com/p/gapdh-antibody-0411<br>https://www.selleckchem.com/products/infliximab.html#:~:text=Infliximab%20(anti%2DTNF%2Dalpha)%20(Remicade%2C%<br>20Remsima,and%20light%20chain%20constant%20regions.<br>https://www.sinobiological.com/antibodies/human-asgr1-10773-mm02<br>https://www.thermofisher.com/us/en/home/life-science/antibodies/invitrogen-antibody-validation.html

Eukaryotic cell lines

Policy information about cell lines and Sex and Gender in Research

Cell line source(s)	The human iPSC line GM25256 (WTC) was obtained from Bruce Conklin at the Gladstone Institute of Data Science and Biotechnology. Additional information can be found at https://www.coriell.org/0/Sections/Search/Sample_Detail.aspx?Ref=GM25256.<br><br>The human iPSC lines CW10001, CW10030, CW10037, CW10152, CW10201 and CW10208 were obtained from the CIRM iPSC Repository available at Fujifilm Cellular Dynamics. Additional information can be found at https://www.fujifilmcdi.com/.<br><br>Primary human hepatocytes (Lot: BMO) were purchased from BioIVT. Additional information can be found at https://bioivt.com/human-cryoplateable-hepatocytes.<br><br>Primary human macrophages were generated from peripheral blood mononuclear cells isolated from healthy volunteers.
Authentication	Human iPSC lines were authenticated using SNP analysis. No authentication was performed for primary human cells.
Mycoplasma contamination	All cells were routinely tested for mycoplasma using Lonza MycoAlert Detection Kit and found to be negative.
Commonly misidentified lines<br>(See ICLAC register)	No commonly misidentified cell lines were used in this study.

Flow Cytometry

Plots

Confirm that:

☒ The axis labels state the marker and fluorochrome used (e.g. CD4-FITC).

☒ The axis scales are clearly visible. Include numbers along axes only for bottom left plot of group (a 'group' is an analysis of identical markers).

☒ All plots are contour plots with outliers or pseudocolor plots.

☒ A numerical value for number of cells or percentage (with statistics) is provided.

Methodology

Sample preparation	Freshly thawed primary human hepatocytes, primary human macrophages detached using Accutase, fully differentiated human iPSC-derived hepatocytes detached using 0.25% trypsin-EDTA and human M1/M0 iPSC-derived macrophages detached using Accutase were incubated with the respective antibodies in PBS including 0.1% BSA and 2 mM EDTA for 20 minutes at 4°C and washed once before flow cytometry analysis.

Instrument	LSRFortessa flow cytometer (BD Biosciences).
Software	LEGENDplex data analysis software (v8.0), FloJo (v10.6.1).
Cell population abundance	No cells were sorted.
Gating strategy	The main cell population was selected based on pulse area (-A) of forward scatter (FSC) and side scatter (SSC). Pulse height (-H) of FSC and SSC was used to select single cells from the main cell population. SYTOX green/red cell death staining was used to select viable cells. Gates for target cells expressing specific markers detected with fluorophore-conjugated antibodies were defined based on unstained cells. 450_50 Violet F-A and 582_15 YG D-A are flow cytometer-specific laser and filter set combinations for fluorophore detection. A representative gating strategy example is shown in Supplementary Fig. 8.

☒ Tick this box to confirm that a figure exemplifying the gating strategy is provided in the Supplementary Information.
